# A prospective cross-sectional study on pharmacovigilance of antipsychotic drugs at a tertiary care teaching hospital

**DOI:** 10.6026/973206300222598

**Published:** 2026-04-30

**Authors:** Parth Jain, Shashi Marko, Lily Dubey, Pawan Gupta

**Affiliations:** 1 Department of Pharmacology, Bundelkhand Medical College, Sagar, Madhya Pradesh, India; 2 Department of Dermatology, Bundelkhand Medical College, Sagar, Madhya Pradesh, India

**Keywords:** Adverse drug reactions (ADRs), causality assessment, WHO-UMC scale, Naranjo scale, antipsychotic drugs, preventability, schumock-thornton scale

## Abstract

Antipsychotic medications are associated with many adverse drug reactions (ADRs) which affect patient compliance, safety and outcomes, making pharmacovigilance important, that by pharmacovigilance is performed to gather data, make decisions and prevent ADRs of antipsychotic medications. Hence, this is a prospective cross-sectional observational study WHO-UMC scale and Naranjo scale was used to assess the causality of the adverse drug reactions with oral antipsychotic medications. Total 61 patients with adverse drug reactions were observed, 168 adverse drug reactions were observed because of 5 medications; according to WHO-UMC scale 52.45% adverse drug reactions were probable/ likely and according to Naranjo scale 52.45% adverse drug reactions were possible. Pharmacovigilance helps us assess adverse drug reactions, it guides our clinical decision making and drug selection, it gives us data which helps us better perform risk-benefit analysis of drug administration ultimately serving patient wellbeing.

## Background:

Pharmacovigilance is an excellent tool to assess the effectiveness and safety of new and pre-existing medications. The role of pharmacovigilance is to detect, evaluate and prevent medicine administration associated safety issues [1]. Data gathered from pharmacovigilance programmes is used to identify public health issues and make policies related to preventing and managing adverse drug reactions. In Israel, a change in the excipient of Eltroxin led to an imbalance of thyroid hormone levels leading to a huge number of adverse drug reactions, mainly in the form of hyper and hypothyroidism [2]. Pharmacovigilance focuses on the pharmaceutical industry, hospitals, clinics, health care centres, medical institutions, pharmacies, drug distributors and everyone involved with the drug industry, either directly or indirectly, such as registered medical practioners, pharmacists and even patients. ADRs are the fourth to fifth leading cause of death in the USA and Europe [3]. One thing that must be kept in mind while performing pharmacovigilance is risk management and risk prevention associated with medicine administration [4]. There are multiple reasons causing ADRs, like faulty manufacturing, change of excipients across batches, medication error, prescription errors, wrong dose, wrong drug combinations, irrational prescription, improper understanding by the patient, improper dispensing by the pharmacists, quacks and unauthorized individuals prescribing drugs without knowledge and understanding [5]. Pharmacovigilance process uses tools like causality assessment, severity and preventability assessment to better understand and manage adverse drug reactions [8]. Treatment discontinuation is a consequence of adverse drug reactions which needs to be studied to prevent therapeutic failure [7].

## Materials and Methods:

This is a Prospective cross-sectional observational study conducted in department of psychiatry and department of pharmacology of Bundelkhand medical college, Sagar Madhya Pradesh. The study was conducted for duration of one and a half years November 2023 to April 2025. This research was envisaged to study ADRs associated with antipsychotic drugs. The primary objective was to record and perform causality assessment of the ADRs observed. The secondary objectives were to determine the frequency of ADRs with each drug prescribed and their preventability.

## Inclusion criteria:

[1] All participants who consent to the study

[2] Participants on one or more than one antipsychotic medication with ADR(s)

## Exclusion criteria:

[1] Participants not prescribed any antipsychotic medication

[2] Participants who do not consent to the study

[3] Participants with severe co-morbidities

## Ethical approval:

Ethical approval was obtained from BMC Institution Ethics Committee on 19-7-2023.

## Statistical analysis:

The data was collected from psychiatry OPD patients on antipsychotic with presenting ADRs. A total of 200 patients were included in the study. WHO-UMC and Naranjo scale were used for causality assessment. Schumock-Thornton criteria were used for preventability assessment. Data analysis for determining chi-square statistics, degree of freedom and p value was performed using Microsoft Excel 2024 version, SPSS V29, online statistics calculators.

## Results:

A total of 61 patients with ADRs were seen and 168 ADRs were recorded with these five antipsychotic drugs when assessed individually and/or in combination with other antipsychotic drugs. The total numbers of ADRs reported with each drug (Table 1 - see PDF) are as follows: Olanzapine - 70 (p = 0.094, χ^2^ = 22.55), Clozapine - 31 (p = 0.598, χ^2^ = 13.06), Aripiprazole - 31 (p = 0.537, χ^2^ = 10.89), Risperidone - 26 (p = 0.745, χ^2^ = 8.5), Quetiapine - 10 (p = 0.986, χ^2^ = 10.36). A total of 70 ADRs were reported with Olanzapine. Chi-square test showed p = 0.094, indicating no statistically significant difference compared to expected values. In the case of Aripiprazole (p = 0.537) and Clozapine (p = 0.598) the result is also not statistically significant and may be due to random variation. On performing the causality assessment, it was found that 24.59% were rated certain in causality, 52.45% were probable/likely, 19.67% were possible, 1.61% were conditional/unclassifiable and 1.63% were unassessable/unclassifiable using the WHO-UMC scale (p = 0.0001, χ^2^ = 53.344, df = 4) (Table 2 - see PDF), meaning that the majority of ADRs were not random and were linked to antipsychotic exposure. With the Naranjo scale (Table 3 - see PDF), the result of causality assessment is as follows: "doubtful" causality was 1.639%, "probable" was 42.62%, "possible" was 52.45% and "definite" was 0% (p = 0.0001, χ^2^ = 58.279, df = 3), giving statistical significance to the relation between antipsychotic exposure and ADRs. Concomitant drugs (Table 4 - see PDF) were prescribed a total of 44 times in these 61 patients (p = 0.00046, χ^2^ = 29.89, df = 9). These drugs are as follows: Multivitamin and related supplements, Trihexyphenidyl, Ondansetron, Divalproex, Lactulose, Fluoxetine, Zolpidem, Lithium, Carbamazepine, Oxcarbazepine and Pantoprazole. Using the Schumock-Thornton criteria, 51 patients had "non-preventable" ADRs, 11 patients had "probably" preventable and 2 patients had "definitely" preventable ADRs (p = 0.0001, χ^2^ = 63.78) (Table 5 - see PDF), meaning that the majority of ADRs were non-preventable. On performing the causality assessment, it was found that 24.59% were rated certain in causality, 52.45% were probable/likely, 19.67% were possible, 1.61% were conditional/unclassifiable and 1.63% were unassessable/unclassifiable using the WHO-UMC scale (p = 0.0001, χ^2^ = 53.344, df = 4) (Table 2 - see PDF), meaning that the majority of ADRs were not random and were linked to antipsychotic exposure. With the Naranjo scale (Table 3 - see PDF), the result of causality assessment is as follows: "doubtful" causality was 1.639%, "probable" was 42.62%, "possible" was 52.45% and "definite" was 0% (p = 0.0001, χ^2^ = 58.279, df = 3), giving statistical significance to the relation between antipsychotic exposure and ADRs. Concomitant drugs (Table 4 - see PDF) were prescribed a total of 44 times in these 61 patients (p = 0.00046, χ^2^ = 29.89, df = 9). These drugs are listed in the table below. Using the Schumock-Thornton criteria, 51 patients had "non-preventable" ADRs, 11 patients had "probably" preventable and 2 patients had "definitely" preventable ADRs (p = 0.0001, χ^2^ = 63.78) (Table 5 - see PDF), meaning that the majority of ADRs were non- preventable.

## Discussion:

Although Olanzapine accounted for the highest proportion of ADRs, this finding should be interpreted cautiously, as it was also the most frequently prescribed antipsychotic in the study population. Therefore, the higher number of reported ADRs may be attributable to greater patient exposure rather than an inherently higher risk profile of the drug [7]. Number of ADRs with risperidone and quetiapine had consistent result to ESQUIRE trial in having no statistical significance, meaning that any difference in ADR counts is likely due to chance, not due to a real effect or risk tied to those drugs, there is no strong evidence that the drugs have a higher or lower rate of ADRs than expected [8, 3]. However, our results differ from larger pharmacovigilance datasets where Clozapine and Olanzapine often emerge as high-signal drugs for metabolic and haematological ADRs [9, 10 and 11]. One reason for these findings is the relatively small sample size, under reporting and local prescribing trends, with Olanzapine use being more frequent in our case. This exposure bias may alter the ADR counts without reaching statistical significance. None of the drugs showed a statistically significant difference in ADR frequency compared to expected rates (p > 0.05), suggesting that observed variations may be due to random chance. Causality assessment further strengthened the link between the observed ADRs and antipsychotic exposure. The WHO- UMC and Naranjo causality assessments classified over half of the ADRs as probable or likely, strengthening the evidence for a causal link between the observed reactions and antipsychotic exposure. These findings are consistent with previous Indian studies [10, 11 and 12], which also reported high proportions of probable ADRs for antipsychotics. This also highlighted the need of using a different rout of administration so as to decrease the occurrence adverse drug reaction [13]. Currently, in our institution pharmacovigilance is being performed by pharmacologists and pharmacology residents as a part of their regular duty, research and thesis process and by doctors aware of the pharmacovigilance procedure. Among the residents working in hospital, awareness for pharmacovigilance is low due to workload, lack of training and standard operating procedures [14, 15]. Making pharmacovigilance a part of the clinical procedure will help in its application in the clinical setting and build awareness [16, 17]. In summary, this study did not find statistically significant differences in ADR frequency between antipsychotics but the high proportion of probable causality rating shows the clinical relevance of pharmacovigilance in psychiatric practice. Limitations of this study are - 1) missing or incomplete ADR documentation), 2) small sample size (limits statistical power), 3) ADR detection depends on spontaneous reporting (mild and moderate ADRs may go unreported or under reported). This study opens the path for a prospective cohort study assessing the relation between antipsychotic drugs and their ADRs.

## Conclusion:

Pharmacovigilance helps us improve patient care. It is important in making decisions while prescribing drugs that may have permanent, disabling, or dangerous ADRs. The data generated helped us understand the ADR profile of different drugs in our institution, which gives an edge by helping us predict, prepare and manage these ADRs. This study helped us understand the prevalence of antipsychotic associated ADRs. Also, it helps compare the ADRs observed at different regions, thereby helping us establish variation among different demographic populations.

## Funding:

None
